# Statin use and peripheral sensory neuropathy: a pilot study

**DOI:** 10.1186/1757-1146-6-S1-O40

**Published:** 2013-05-31

**Authors:** Brenton West, Cylie Williams, Elise Jilbert, Alicia James, Terry Haines

**Affiliations:** 1 Peninsula Health Podiatry Department Frankston, VIC 3199, Australia; 2 Southern Health Allied Health Clinical Research Department, Cheltenham, VIC 3192, Australia; 3 Monash University, Department of Physiotherapy, Frankston, VIC, 3199, Australia

## Background

Peripheral sensory neuropathy is a neurological deficit resulting in decreased detection of sensation through the peripheral nervous system. Statins are a widely used medication and there has been some debate of association with their use and the presence of peripheral sensory neuropathy. This pilot study aimed to test the sensory perception of participants with long term statin use and compare these results to their peers who were not taking statins.

## Methods

30 participants were recruited for this study and equally divided into a statin and non-statin group. Healthy participants were screened by their medical and medication history, Australian Type 2 Diabetes Risk assessment and random blood glucose level. An assessor who was blinded to the participant group conducted sensory assessments using the 10g monofilament and neurothesiometer.

## Results

There was no difference in monofilament testing results between the groups. The statin group was less sensate at the styloid process (p=0.031) and medial malleolus (p=0.003) than the control group. Results at the hallux were not statistically significant (0.183).

## Conclusion

This result is suggestive of a potential association between long term statin use and the development of peripheral sensory neuropathy. As statins are a life saving medication, careful consideration should be applied to these results and further research be conducted to determine if these results are applicable to larger populations. Prescribers of statins should be aware and considerate of the potential decrease in sensory perception and monitor their foot health accordingly.

